# Editorial: Urgent injury and violence-related public health threats: the role of social determinants in cross-cutting injury and violence across the lifespan

**DOI:** 10.3389/fpubh.2024.1457030

**Published:** 2024-08-14

**Authors:** Ursula Kelly, Joseph Carpenter, Sangmi Kim, Jill Woodard, Dabney P. Evans

**Affiliations:** ^1^Nell Hodgson Woodruff School of Nursing, Emory University, Atlanta, GA, United States; ^2^School of Medicine, Emory University, Atlanta, GA, United States; ^3^Rollins School of Public Health, Emory University, Atlanta, GA, United States

**Keywords:** injury, violence, health inequities, social determinants of health (MeSH), prevention, addiction, firearms, public health policies

## Introduction

Our goals for this Research Topic are to disseminate science that advances our understanding of the ways in which social determinants of health (SDOH) impact injury and violence and to identify best practices for addressing these urgent public health priorities. We grouped articles into three settings: clinical, community, and policies; urgent public health priorities include the opioid epidemic, gun violence, adverse childhood experiences (ACE), interpersonal violence, and suicide. The articles are relevant to readers in different settings, disciplines, and focus areas, given the cross-cutting nature of injury and violence across the lifespan.

## Clinical

SDOH influence health outcomes as well as the provision of health care. Clinical settings and individual clinical encounters are avenues to address SDOH-related health disparities via enhanced screening, trauma-informed care, and targeted education. The clinical setting-based articles in this Research Topic examined the role of SDOH in a broad range of violence- and injury-related subjects across hospital and outpatient settings.

ACEs and exposure to violence are associated with the subsequent development of chronic health conditions. Two author groups explored these relationships within specific populations: Afzal et al. found that cumulative ACEs are a strong predictor of chronic health conditions, independent of other SDOH among U.S. adults. Sales et al. reported that most people living with HIV they surveyed reported a history of ACEs or intimate partner violence (IPV). Both groups called for enhanced screening for ACEs or IPV, particularly among populations not traditionally targeted for screening, for example, asking male people living with HIV about IPV as an avenue for early intervention. Importantly, Sales et al. noted that the prevalence of ACEs and IPV was so high that the best practice may be to employ a universal trauma-informed approach to care, an approach that is likely beneficial to other/all patient groups.

Trauma-informed care models seek to level power dynamics between patients and providers, emphasizing collaboration and transparency and avoidance of re-traumatization. Through a series of vignettes, Febres-Cordero et al. described the stigmatization of substance use and assumptions of “drug seeking behavior” by nurses. Here, stigma is seen as an abuse of authority, violating patients' rights of autonomy, dignity, and self-advocacy. Harm reduction is framed not simply as a treatment alternative but instead as an issue of health equity, social justice, and human rights. A call is issued to practice trauma-informed care in patients with pain or a history of substance use, echoing the call of Sales et al. for a universal approach.

Patients hospitalized following injuries encounter many health disparities based on SDOH. Giordano et al. used Medicaid claims data to demonstrate racial and ethnic disparities in opioid prescribing after orthopedic injury, highlighting biased and inequitable assessment and treatment of pain in Black and Hispanic individuals, and a need for scalable interventions to address these disparities. In an excellent example of community-engaged research, Zeidan et al. interviewed Spanish-speaking immigrants admitted for work-related injuries. After identifying a startling level of comfort working in hazardous environments, often with little support from employers, the authors introduced immigrant status by itself as a risk factor for injury, an often-under recognized SDOH. They went on to offer an opportunity for targeted intervention—specifically to offer targeted “Know Your Rights” training during hospitalization to decrease the chance of re-injury.

## Community

Community is conceptualized as the physical and social environments inhabited by individuals that shape both their risk of injury and violence and the resources to protect against or mitigate their risk. In this Research Topic, four risk/protective factors at the community level were highlighted: hospital/community-based organization (CBO) partnerships, evidence-based in-home services, i.e., SafeCare, and community outreach; and more broadly, neighborhoods' quality of resources and conditions to prevent IPV and gun violence. The SDOH identified by the authors span the SDOH categories of Neighborhood and Built Environment and Social and Community Context. Accordingly, the authors of these community-based articles described cross-sector problems and approaches, a departure from the prevalent community intervention model in which individual institutions and organizations use their specific resources and strengths to serve their populations of interest.

The authors identified a critical need for strengthened cross-institutional partnerships, education (for the public and professionals), and outreach to community members to amplify the effectiveness of violence prevention efforts through more integrative approaches. In that regard, Evans et al. called for more coordinated care between hospitals and CBOs for IPV survivors receiving care in an Emergency Department. Specifically, they suggested cross-training among personnel in hospitals and CBOs, warm handoff, co-location of services (domestic violence service organization staff housed in the emergency room), and improved communication between involved organizations and institutions. In addition, Osborne et al. discussed the role of an evidence-based in-home behavioral parenting program originally targeting child maltreatment (SafeCare) in pediatric firearm injury prevention. They suggested developing formal guidance or curricula for firearm secure storage counseling tailored to SafeCare providers and training SafeCare providers to improve their self-efficacy in discussing firearm safety at home. Moreover, healthcare providers are apt to provide parents with guidance on firearm safe storage to prevent unintentional and self-inflicted intentional injury in children. Fraser Doh et al. underlined the effectiveness of community outreach to educate and counsel parents and distribute safe storage devices, which was well-accepted by parents, demonstrated by a high percentage of using the provided safe storage device at follow-up.

Importantly, the availability and accessibility to such community resources are heavily influenced by the broader environments where individuals live, work, play, and learn. As Reddy discussed, pediatric firearm injury is concentrated in disadvantaged neighborhoods highly populated with racial/ethnic minorities (e.g., Black and Hispanic populations). A wide range of neighborhood conditions impact violence, such as green space, walkability, house vacancy, presence of early childhood education centers, proximity to schools, toxic exposures, food insecurity, employment, and poverty, among others, whose distribution is deeply rooted in structural racism (e.g., redlining, segregation), and hence, requiring policy change.

## Policies

Policies—broadly understood as codified laws, principled plans of action, and written procedures—are important structural drivers of SDOH that can lead to health disparities. The submissions in this Research Topic identify necessary improvements in state and federal laws (e.g., 988 suicide hotline) and include calls to action for policy development to effectively prevent violence-related morbidity and mortality, which disproportionately affect disempowered populations, that is, unequal access to opportunities for health and safety. Notable themes across the policy-related articles are: (1) improved problematization and clearer definitions of types and causes of violence; (2) improved injury and violence surveillance systems and prevention; and (3) proposed systems improvements. Ziminski, in a social problem analysis of two firearm-related laws, identified the lack of social problematization of firearm violence, including its causes, context, and contributing factors. Similarly, Lewis et al., in their commentary on femicide, cited the need for a clear and codified definition of femicide in U.S. law and called for improved surveillance systems and use of evidence-based practices by law enforcement and criminal justice systems.

Surveillance systems are an important means to assess population health, allow for the identification of disproportionately affected populations, and inform interventions tailored to the population's needs. Both Ziminski and Lewis et al. called for improved and expanded violence-related surveillance systems and disaggregated data to enable the allocation of prevention efforts and resources toward the most affected communities and population groups. These calls are supported by Mulugeta et al., who found increases in pediatric firearm injury after the passage of a Georgia state law legalizing permit-less concealed carry of a firearm; the most affected population was Black and publicly insured boys who were injured through assault and unintentional shootings. The role of intersecting identity-based vulnerabilities was a thread through most policy articles.

Several authors explicitly addressed the role of social policy and systems on injury and violence. Baker and Sorenson examined the effects of the enactment of the national 988 suicide hotline in Georgia and noted that state context is an important consideration in the analysis of federal policy implementation. They observed a behavioral health workforce shortage and a lack of accessible and available healthcare in Georgia—a state that rejected Medicaid expansion. These authors' policy recommendations included sustainable behavioral and mental health federal funding (e.g., through SAMHSA subsidies and tax revenue) and strengthening health systems. Similarly, Jahangir et al. examined a Temporary Assistance for Needy Families (TANF) diversion program aimed at IPV prevention. They observed the protective effects of the diversion program and concluded that the policy was functioning. However, they also described how the program disincentivized people in need from seeking public assistance, creating barriers to other TANF benefits. The works of these authors highlight the limitations and sometimes unanticipated negative consequences of policies designed to support people in need of social support (e.g., those in crisis and survivors of IPV). The processes of policy development and implementation need to include careful consideration of how policies cause downstream health disparities.

## Conclusion

SDOH and the urgent public health crises addressed in this Research Topic—ACEs, the opioid epidemic, gun violence, interpersonal violence, and suicide—are inextricably linked. Despite the complexities of SDOH and injury and violence, there are some clear and important takeaways from these articles.

Social resources are inequitably shared, leaving those without these resources disproportionately affected by violence compounded by multiple barriers to having their social and health care needs met. Public policies shape and determine the inequitable distribution of social resources; these public policies at local, state, and federal levels are themselves shaped and determined by political influence and reflect both persisting and transient social values that do not necessarily align with public health needs. To address these needs effectively, a shared perspective and meaningful collaboration across health care institutions, CBOs, and public health entities must replace the current approach to injury and violence prevention that is compartmentalized by their type. At the same time, it is critical to center the experiences and needs of individuals affected by injury and violence to inform and guide clinical interactions (trauma-informed care) and develop evidence-based community interventions and public policy. Public policy, arguably the most upstream driver of SDOH, requires meaningful input from the field of public health to maximize the *public's health* and eliminate health disparities. Thus, we strongly urge legislation to provide on-going robust funding for U.S. federal agencies charged with protecting the public's health and improving individual and public health related to addiction, mental illness, and violence, e.g., the National Center for Injury Prevention and Control in the CDC, the Substance Abuse and Mental Health Services Agency, and the National Institutes of Health. Additionally legislative priorities are needed to reduce access to lethal means and improve access to lifesaving means to prevent suicide, homicide, opioid overdose, and family violence. Likewise, the need for research to develop evidence on injury-related Research Topics is urgent. Existing NIH-supported research on injury is limited and additional funds are needed to advance scientific knowledge to inform public policies. Two important research areas are cross-sector (health care, social services, public health, and community groups) studies to address SDOH as they relate to the opioid epidemic, gun violence, ACEs, interpersonal violence, and suicide; and implementation science to apply existing evidence to these urgent public health threats, particularly targeting both risk and protective factors.

## Author contributions

UK: Writing – original draft, Writing – review & editing. JC: Writing – original draft, Writing – review & editing. SK: Writing – original draft, Writing – review & editing. JW: Writing – original draft, Writing – review & editing. DE: Writing – original draft, Writing – review & editing.

